# Estimates of the burden of illness for eight enteric pathogens associated with animal contact in Canada

**DOI:** 10.1017/S0950268817002436

**Published:** 2017-11-23

**Authors:** R. MURRAY, J. TATARYN, K. PINTAR, M. K. THOMAS

**Affiliations:** Centre for Foodborne, Environmental and Zoonotic Infectious Diseases, Public Health Agency of Canada, Guelph, Ontario, Canada

**Keywords:** Enteric bacteria, epidemiology, water-borne infections

## Abstract

Enteric pathogens are commonly known to be transmitted through food or water; however, contact with animals is another important transmission route. This study estimated the annual burden of illness attributable to animal contact for eight enteric pathogens in Canada. Using data from a Canadian expert elicitation on transmission routes, the proportion of enteric illnesses attributable to animal contact was estimated for each pathogen to estimate the annual number of illnesses, hospitalizations and deaths in Canada. For each estimate, a mean and probability intervals were generated. Of all illnesses caused by these eight pathogens, 16% were estimated attributable to animal contact. This estimate translates to 86 000 (31 000–166 000) illnesses, 488 (186–890) hospitalizations and 12 (2–28) deaths annually for the eight pathogens combined. *Campylobacter* spp. is the leading cause of illnesses annually, with an estimated 38 000 (14 000–71 000) illnesses occurring each year, followed by non-typhoidal *Salmonella* spp. (17 000, 6000–32 000). The majority of hospitalizations were attributable to non-typhoidal *Salmonella* spp. (36%) and *Campylobacter* spp. (31%). Non-typhoidal *Salmonella* spp. (28%) and *Listeria monocytogenes* (31%) were responsible for the majority of the estimated deaths. These results identify farm animal and pet/pet food exposure as key pathways of transmission for several pathogens. The estimated burden of illness associated with animal contact is substantial.

## INTRODUCTION

Enteric zoonotic pathogens are commonly known to be transmitted through food or water; direct or indirect contact with an animal is another key route of transmission [1–3]. The burden of enteric (acute gastrointestinal) illness associated with contact with farm animals, wildlife, domestic pets and their environment is not well known in Canada. Several outbreaks have highlighted pets as an important route of transmission. Salmonellosis outbreaks linked to pet food and treats [4, 5], live poultry, reptiles and amphibians, and rodents have been reported in Canada [6–8] and the United States (USA) [9–15] in recent years. Outbreaks of verotoxigenic *Escherichia coli* (VTEC *E. coli*) associated with petting zoos have been identified several times [16–19]. In particular children are at a higher risk from this transmission route, given their behaviors (close proximity with pets, petting zoos, more likely to put hands in their mouth, etc.) and developing immune systems [16, 20–23]. Case–control studies have identified an increased risk of illness from exposures to animal contact on farms for *Campylobacter* spp., non-typhoidal *Salmonella* spp., VTEC O157 [20, 24–26] and specifically contact with cattle for *Cryptosporidium* infections [27].

Enteric illnesses are underascertained by public health surveillance systems because of underdiagnosis and under-reporting [28]. In order to more accurately estimate the burden of illness associated with animal contact, it is necessary to account for underascertainment and to estimate what proportion of illnesses result from animal transmission, as pathogens rarely are exclusive to one transmission route. To account for the underascertainment at each level of the public health surveillance system (i.e. case seeking medical care, submitting a sample, the sample being tested and found positive, and the positive test result being reported), pathogen-specific multipliers have been used. Estimates related to foodborne illness, and the associated hospitalizations and deaths in Canada and the USA, have been developed using this method of developing underascertainment multipliers and incorporating pathogen-specific source attribution estimates [29, 30].

The USA has estimated the burden of illness (using underascertainment multipliers as described above) associated with animal contact for seven key enteric pathogens and found that 14% of illnesses are attributed to contact with animals and their environments [31]. Reported case data from the province of Ontario, Canada, identified contact with animals as the primary source of exposure for nearly 20% of the reported cases due to 14 pathogens [32]. Estimating transmission routes for enteric pathogens is difficult due to the lack of data. Expert elicitations have been conducted in Canada and internationally to estimate the proportion of enteric illnesses attributed to different transmission routes including foodborne and animal contact [33–36].

The objective of this study is to estimate the number of illnesses, hospitalizations and deaths in Canada related to animal contact, using estimates of enteric illness in Canada and the proportion of cases attributed to animal-related contact based on an expert elicitation [37]. The expert elicitation asked 31 Canadian experts to estimate the proportion of cases of 28 pathogens transmitted by five main transmission routes (food, water, animal contact, person-to-person and other) and select subcategories of the food, water and animal contact routes [35, 37]. The current study builds upon the elicitation results by estimating the number of cases related to the animal contact route and subcategories of domestic pets, farm animals and wildlife specifically. These burden estimates can be used to better describe and increase awareness of this public health problem, and inform advocacy, education and further research activities, to prevent and reduce enteric illnesses associated with animal contact.

## METHODS

This study estimated the annual number of animal-related illnesses, hospitalizations and deaths related to eight key pathogens (*Campylobacter* spp., non-typhoidal *Salmonella* spp., *Giardia* sp., *Cryptosporidium* spp., VTEC O157, VTEC non-O157, *Yersinia enterocolitica* and *Listeria monocytogenes*). Pathogens selection for this study was, in part, based on the seven included in the US study [31] with the addition of *Giardia* sp., which was selected based on the Canadian expert elicitation attributing 14% of giardiasis to animal contact. Other zoonotic enteric pathogens were not included due to the lack of available data.

Data focused on the 2000–2010 time period for the underascertainment multipliers and laboratory-confirmed cases, hospitalizations and deaths and was based on the approximate Canadian 2006 census population (32 500 000), to follow the methods published for the burden of foodborne illness in Canada [28, 30]. Details on specific data sources for the illness estimates and proportion of illness caused by animal contact are provided below. The reported and estimated values used in this analysis are provided as reference in Table 1. All estimates reflect the 2000–2010 time period.

**Table 1.** Reported and estimated annual number of illnesses, hospitalizations and deaths for eight enteric pathogens, Canada

	Illnesses		Hospitalizations		Deaths	
Pathogen	Reported annually (CNDSS 2000–2010)	Estimated mean annual number (accounting for underascertainment)	Reported annually (CIHI-HMDB 2007–2010)	Estimated mean annual number (accounting for underascertainment)	Reported annually (CIHI-HMDB 2007–2010)	Estimated mean annual number (accounting for underascertainment)
*Campylobacter* spp.	10 037	272 930	598	1074	6	11
*Salmonella* spp., non-typhoidal^a^	5774	150 716	857	1565	16	29
*Giardia*^a^	4374	177 917	132	467	3	10
*Cryptosporidium* spp.	723	35 092	35	124	0·4	1
*Yersinia enterocolitica*	975	38 333	44	119	2	4
VTEC O157	883	17 715	208	340	7	11
*Listeria monocytogenes*	131	226	124	190	29	44
VTEC non-O157	NA	NA	22	305	1	2

^a^Values for reported hospitalizations and deaths from CIHI-HMDB for *Salmonella* spp., non-typhoidal and *Giardia* are based on the 2000–2010 data.

### Estimating total illnesses, hospitalizations and deaths

The methods for estimating the number of domestically acquired foodborne illnesses are described in detail elsewhere [28]. In brief, using this approach of estimating the number of domestically acquired cases by pathogen data on the number of laboratory confirmed cases for seven of the eight pathogens were obtained from national surveillance systems for the time period 2000–2010. These values were then adjusted for under-reporting (i.e. laboratory confirmed but not reported to local/provincial/territorial public health and national surveillance systems) and underdiagnosis (i.e. those who do not seek medical care, sample is not submitted, tested or found positive for causative pathogen) based on data from Canadian National Studies on Acute Gastrointestinal Illness (NSAGI) population, laboratory and public health reporting surveys [38–42]. An alternative approach was used for estimating VTEC non-O157 cases, which is not routinely identified and reported in Canada. For VTEC non-O157 cases, a ratio (1 VTEC O157:1·6 VTEC non-O157, based on literature [43]), relative to the estimate of VTEC O157 cases was used [28, 30]. The methods for estimating the number of domestically acquired foodborne hospitalizations and deaths are described in detail elsewhere [30]. In brief, the methods for estimating hospitalizations and deaths for these eight enteric pathogens relied on the number of hospitalizations and deaths for each pathogen reported in the Canadian Institute for Health Information Hospital Morbidity Database (during the 2000–2010 time period, for certain pathogens only a subset of this time period was available, Table 1) [30, 44]. These values were then adjusted for under-reporting (in the hospital database) and underdiagnosis (i.e. sample is not submitted, tested or found positive for causative pathogen). International travel-related illnesses, hospitalizations and deaths were excluded by subtracting the pathogen-specific proportion that is travel-related.

### Proportion of illnesses caused by animal contact

The pathogen-specific proportion of illness attributable to animal contact was estimated using findings from a recently conducted Canadian enteric illness transmission expert elicitation [35]. As reported in the Methods section of the Canadian expert elicitation study, 31 experts estimated the proportion of illnesses caused by 28 pathogens transmitted via major transmission routes (foodborne, waterborne, animal contact, person-to-person and other) and for select subcategories of those major routes, at the point of pathogen consumption. The elicitation consisted of a snowball expert recruitment and collection of background information on experts, an initial online elicitation survey followed by a results discussion and a second online elicitation as an opportunity for experts to modify their initial responses based on the discussion. Animal transmission was defined as an illness transmitted by exposure to animals, i.e. personal contact (hand or mouth) with animal/pet feed, animal/pet fur/coats, saliva or feces (Table 2). The proportion of illnesses from contact with domestic pets, farm animals and wildlife were estimated for non-typhoidal *Salmonella* spp., *Campylobacter* spp., *Giardia* sp., VTEC O157 and *Y. enterocolitica* based on the definitions in Table 2 [37]. As previously described, triangular probability distributions were built from the expert estimates for each transmission route and pathogen, using @Risk software (Version 6·1·2; Palisade Corporation, Newfield, NY, USA) from best estimate (most likely) and 5th and 95th percentile values. These were then combined into cumulative distributions, using Monte Carlo simulation with 10 000 iterations [35, 37].

**Table 2.** Definitions of animal contact and subcategory transmission used in expert elicitation survey, Canada 2014 [35, 37]

	Definitions
Animal contact	Illness transmitted by exposure to animals, i.e. personal contact (hand or mouth) with animal/pet feed, animal/pet fur/coats, saliva or feces
Animal contact subcategories	
Domestic pets/companion animals	Household pets including cats, dogs, rabbits, reptiles and birds
Farm animals	Including cattle, horses, sheep and exposure to the same animals in petting zoos, fairs and animal exhibits
Wildlife	Including dead or live deer, foxes, crows, rats, raccoons, birds

### Analysis

The median and 90% credible intervals for the proportion of illnesses due to animal contact and transmission subcategories, by pathogen, as defined by the Canadian expert elicitation, were entered into individual pathogen models as a PERT distribution, where the median and 90% credible interval values from the expert elicitation were used as the inputs for mean and the upper and lower bounds of the PERT distribution. The @RISK add-in for Microsoft Excel was used, with 100 000 iterations to generate a mean and 90% probability intervals (PIs) for estimates.

## RESULTS

These eight pathogens account for 528 279 domestically acquired enteric (acute gastrointestinal) illnesses each year in Canada of which an estimate of 84 751 (90% PI 52 952–123 985 were related to animal contact (Table 3)), reflecting approximately 16% of all domestically acquired enteric illnesses due to these pathogens. An annual incidence rate of 261 illnesses per 100 000 Canadians is attributed to animal contact for these eight pathogens. *Campylobacter* spp. is estimated to cause 38 007 (90% PI 14 064–71 600) illnesses each year, the greatest proportion (45%) of the total animal-related illnesses estimated. This is followed by 17 009 (90% PI 6137–32 392) non-typhoidal *Salmonella* spp. (20%) and 16 872 (90% PI 5886–31 928) *Giardia* spp. illnesses (20%).

**Table 3.** Estimated number of illnesses attributed to animal contact for eight enteric pathogens, Canada

Pathogen	Proportion attributable to animal contact (90% CI) [35]	Mean number of illnesses attributable to animal contact (% of total)	90% PI (low)	90% PI (high)	Mean number of hospitalizations attributable to animal contact (% of total)	90% PI (low)	90%PI (high)	Mean number of deaths attributable to animal contact (% of total)	90% PI (low)	90% PI (high)
*Campylobacter* spp.	15·9 (3·5–42·8)	38 007 (45)	14 064	71 600	152 (31)	60	268	1·5 (12)	0·2	4
*Salmonella* spp., non-typhoidal	12·7 (3·0–37·9)	17 009 (20)	6137	32 392	177 (36)	67	320	3 (28)	0·4	7
*Giardia* sp.	13·9 (2·0–35·6)	16 872 (20)	5886	31 928	44 (9)	15	84	1 (8)	0·0	3
*Cryptosporidium* spp.	23 (4·9–57·1)	6305 (7)	2201	12 861	22 (5)	7	47	0·2 (2)	0·0	1
VTEC non-O157	12·3 (2·5–33·4)	4017 (5)	1130	8722	37 (8)	13	70	1 (8)	0·2	3
*Yersinia enterocolitica*	6·7 (0·06–19·3)	2522 (3)	592	3291	8 (2)	2	15	1 (9)	0·4	2
VTEC O157	9·6 (3·6–17·5)	1678 (2)	738	5083	32 (7)	17	52	0·3 (2)	0·0	1
*Listeria monocytogenes*	6·5 (0·05–26·1)	19 (0)	4	39	16 (3)	4	33	4 (31)	1	8
Total		84 751	52 952	123 985	488	326	676	12	6	17

Illness from these eight pathogens associated with animal contact was estimated to be related to 488 (90% PI 326–676) hospitalizations and 12 (90% PI 6–17) deaths (Table 3). Non-typhoidal *Salmonella* spp. is estimated to cause the most hospitalizations (177; 90% PI 67–320), followed by *Campylobacter* spp. (152; 90% PI 60–268), combining for 67% of all hospitalizations. Non-typhoidal *Salmonella* spp. accounted for the greatest number of deaths (3; 90% PI 0·5–7) along with *L. monocytogenes* (4; 90% PI 1–8).

Contact with farm animals was the most common cause of animal-associated illnesses, representing almost 42 608 (90% PI 24 219–66 780) illnesses and over half of the illnesses for the five pathogens for which subcategories were estimated (*Campylobacter*, non-typhoidal *Salmonella* spp., *Giardia* sp., VTEC O157 and *Y. enterocolitica*) (Table 4). An estimated 22 333 (90% PI 7829–43 767) campylobacteriosis illnesses were estimated to be from farm animal contact, *Campylobacter* spp. also accounted for the highest number of illnesses associated with both domestic pets (10 866, 90% PI 3301–22 967) and wildlife (6517 illnesses, 90% PI 1450–15 092). Of the 25 754 (90% PI 13 955–41 139) annual illnesses associated with domestic pets, for each of the non-typhoidal *Salmonella* spp. and *Giardia* sp., about 7000 illnesses were estimated to be related to domestic pets. VTEC O157 and *Y. enterocolitica* illnesses were predominately associated with farm animal contact, with an estimated 1392 (90% PI 492–2735) and 1926 (90% PI 562–3893) illnesses, respectively, each year.

**Table 4.** Estimated number of illnesses attributed to subcategory routes of animal contact for five enteric pathogens, Canada

	Domestic pets/companion animal contact				Farm animal contact				Wildlife contact			
Pathogen	Proportion attributable (90% CI) [37]	Mean number of illnesses attributable (% of total)	90% PI (low)	90% PI (high)	Proportion attributable (90% CI) [37]	Mean number of illnesses attributable (% of total)	90% PI (low)	90% Pl (high)	Proportion attributable (90% CI) [37]	Mean number of illnesses attributable (% of total)	90% Pl (low)	90% Pl (high)
*Campylobacter* spp.	27·5 (8·0–53·6)	10 866 (42)	3301	22 967	57·6 (35·1–87·0)	22 333 (52)	7829	43 767	14·9 (1·2–41·2)	6517 (63)	1450	15 092
*Salmonella* spp., non-typhoidal	39·8 (11·8–75·9)	7001 (27)	2099	14 864	52·6 (21·3–87·6)	9049 (21)	2914	18 497	7·6 (1·9–13·3)	1293 (13)	388	2721
*Giardia* sp.	42·5 (17–66·8)	7136 (28)	2261	14 465	48 (15·8–73·4)	7908 (19)	2480	16 096	9·5 (0·8–30·9)	2163 (21)	675	4508
*Yersinia enterocolitica*	5·3 (3·4–7·3)	662 (3)	128	1599	83 (76·8–89·1)	1926 (5)	562	3893	11·7 (6·5–16·9)	117 (1)	23	280
VTEC O157	23·3 (1·8–62·6)	89 (0)	30	179	72·5 (31·7–95·8)	1392 (3)	492	2735	4·2 (0·4–10·7)	196 (2)	65	399
Total		25 754	13 955	41 139		42 608	24 219	66 780		10 286	4569	19 170

## DISCUSSION

These are the first Canadian estimates of illness attributed to animal contact, accounting for under-reporting and underdiagnosis of illnesses. This study contributes to the development of an understanding of the overall burden of enteric illness in Canada. To date, estimates of foodborne illness [28] and acute gastrointestinal illness associated with drinking water [45, 46] have been completed. This is the first study to estimate illness specifically associated with subcategories of animal contact for farm animals, pets and wildlife. These estimates provide a relative comparison of transmission pathways and dominant sources of enteric illness, which is critical when identifying public health priorities, designing effective interventions, and providing evidence to inform policy and regulatory decision-making at the local, provincial and federal level in Canada. In addition, burden studies help to identify priority pathogens of concern domestically, and knowledge gaps for further research.

Comparing the same seven pathogens included in the US study [31], more illnesses were associated with animal contact in Canada than in the USA, estimating 209 cases per 100 000 Canadians *vs*. an estimated 149 cases per 100 000 in the USA [47]. This is likely due to the differences in total illness estimates as well as generally lower proportions of illness estimated to be via animal transmission in the USA, which relied primarily on case–control studies and outbreak summaries to inform the US inputs. The values used for the proportion of illness attributed to animal contact in Canada from the expert elicitation are within the range of values reported in international studies from the USA, Australia and the Netherlands [31, 33, 34, 36] as well as Canadian studies, using surveillance data and reported exposures [32, 48, 49] (Table 5).

**Table 5.** Comparison of the estimated proportion of domestic cases (and credible intervals (CI)) for eight enteric pathogens attributed to animal contact in previously published Canadian and International studies

	Expert elicitation				Other^a^	Canadian epidemiological studies on reported cases		
	Canada [35]	Australia [33]	The Netherlands [34]	WHO/FERG- subregion A [36]	USA [31]	Ontario, Canada [32]	Canada FoodNet Site [49]	Canada FoodNet Site [48]
Pathogen	Median (90% CI)	Median (95% CI)	Mean (95% CI)	Median (95% CI)	% (range)	Mean	Mean	Mean
*Campylobacter* spp.	15·9 (3·5–42·8)	10 (2–10)	19 (0–60)	11 (0–37)	17 (9–29)	26·9	16·7	20
*Salmonella* spp., non-typhoidal	12·7 (3–37·9)	4 (1–9)	9 (0–19)	10 (0–39)	11 (6–20)	15·2	10·7	21·3
*Giardia* sp.	13·9 (2·1–35·6)	–	11 (0–20)	14 (0–41)	–	14·2	9·8^b^	–
*Cryptosporidium* spp.	23·0 (4·9–57·1)	–	13 (5–19)	10 (1–42)	16 (9–28)	47·2	9·8^b^	–
VTEC non-O157	12·3 (2·5–33·4)	–	–	–	8 (4–15)	–	–	–
*Yersinia enterocolitica*	6·7 (0·6–17·5)	–	–	–	1 (0·5–2)	15·5	–	–
VTEC O157	9·6 (3·6–17·5)	17 (2–35)	21 (0–76)	13 (0–41)	6 (3–11)	18·4^c^	19	9·4
*Listeria monocytogenes*	6·5 (0·5–29·6)	1 (0–3)	5 (0–13)	–	1 (0·5–2)	8·3	–	–
							

a Data sources identified in the USA include Foodnet case–control studies for *Campylobacter* spp., STEC O157, *Listeria monocytogenes*, *Salmonella* spp., non-typhoidal and *Cryptosporidium* spp. Additionally outbreaks were used for STEC O157, STEC non-O157 and *Salmonella* spp., non-typhoidal. There were limited data for *Yersinia enterocolitica*.

^b^*Giardia* sp. and *Cryptosporidium* spp. combined.

^c^All VTEC combined.

This study highlights farm and animal/pet food exposure as an important pathway for illness transmission. Illness associated with farm animals may occur from occupational exposures, such as *Campylobacter* spp. infection among workers at poultry-processing plants [50, 51], non-typhoidal *Salmonella* spp. infection following contact with baby chicks [52] or visiting a petting zoo [2]. Reptiles and related feeder rodents may be responsible for a substantial portion of the pet-related *Salmonella* spp. illnesses estimated [1, 7, 22, 53]. It was estimated in the USA that 6% of all sporadic *Salmonella* infections may be attributable to reptiles or amphibians [54]. While younger puppies may contribute to *Campylobacter* infections [55], pet food has also been found to be a concern for exposure to enteric pathogens [56, 57].

Canadians’ exposure to various animals and pathogen prevalence is an important context to understanding public health risk related to the animal contact transmission route. A Canadian 2015 population study, estimated in the past 7 days that 63·4% of Canadians have ‘any contact with animals, animal waste, habitat or food’, 6·9% visit a farm or barn, and 1·1% and 1·3% visit any petting zoo or an agricultural fair, respectively [58]. The likelihood of enteric pathogen transmission from household pets may be lower compared with an encounter with farm animals; the higher frequency of pet contact [59] would suggest this as an important potential route of transmission.

The prevalence of *Campylobacter* spp. has been reported to be approximately 6·5% for petting zoo animals and 24·7% for household pets [60]. Both of these animal sources have a generally lower prevalence compared with FoodNet Canada data from farms for swine (85%), beef cattle (78%) and dairy cattle (79%) [61]. The prevalence of non-typhoidal *Salmonella* spp. detected in animals on farms (swine, broiler chickens, beef and dairy cattle) by the FoodNet Canada surveillance in sentinel sites across Canada was generally lower than *Campylobacter* spp. [61].

Less is understood about wildlife-associated transmission; based on our study findings, the burden associated with wildlife for all pathogens is notable and the estimated illness associated with *Campylobacter* spp. and non-typhoidal *Salmonella* spp. is considerable. Wild birds may be a primary source of these estimated illnesses as identified as a source of transmission in the United Kingdom (UK) and France [62–64]. It is estimated in the UK that about 10 000 illnesses may be associated with wild birds each year [62]. Garden birds, playgrounds and beach sand activities may be environments where wild birds and human behavior intersect causing illness [65, 66]. Rural wildlife exposure through hunting is also a potential route of transmission and there is evidence that exposure to deer and wild boars may be a source for shiga toxin-producing *E. coli* and *Y. enterocolitica* [67–69].

Limitations of these enteric illness estimate models and the expert elicitation study have been discussed elsewhere [28, 30, 35, 37]. General limitations concerning uncertainty of illness estimate models and potential bias of expert elicitations apply (recruitment, elicitation tool, question framing, methodology and analysis) and have also been discussed elsewhere [70–72]. These estimates reflect illnesses for the time period of 2000–2010 and recent changes in rates of illness for pathogens, such as the reported decrease in *E. coli* O157 cases in Canada in recent years [73] are not reflected in these results and should be considered when interpreting. Sources of information related to the attribution of illness to the animal/pet contact transmission route are limited.

Selection of pathogens to be included may have some limitations as transmission of VTEC non-O157, *Y. enterocolitica*, *L. monocytogenes* and *Giardia* sp. via animal contact is less well known. Contact with farm and domestic animals has been identified as a risk factor for VTEC non-O157 [74, 75] and *Y. enterocolitica*, respectively [76]. While the evidence is less clear for *L. monocytogenes*, transmission from animal to human is plausible, as *Listeria* has been identified in pet food [77], urban poultry flocks [78] and at least one study identified living on a cattle farm as an increased risk of listeriosis [79]. Other countries have also estimated that a small proportion of listeriosis cases may be transmitted via animal contact [31, 33, 34] (Table 5). Animal contact transmission of *Giardia* sp. may be relatively uncommon as current molecular epidemiological data suggests that animals are more often infected with species-specific assemblages that do not cause disease in humans [60, 80–82]. Molecular characterization of *Giardia* sp. in patients in Northern Canada found suggestive zoonotic transmission [83]. Furthermore, animal contact has been implicated in three reported giardiasis outbreaks in a review of *Giardia* sp. outbreaks in the USA (1971–2011), associated with rabbits at a petting zoo, cattle at a farm and a pet reptile at a long-term care facility [84].

The approach used in this study, which is similar to the US approach [31], assumes that the disease severity and frequency with which cases are underdiagnosed are independent of the mode of transmission. In addition, we estimated the overall pathogen-specific proportion of illnesses attributable to animal contact; the proportion of illnesses attributable to animal contact may vary by age because of the differences in exposures and behaviors [31]. The hospitalizations and death estimates may therefore be conservative, as they do not reflect a potential increased representation of illness in children due to this transmission route. The role of sick *vs*. healthy animals and immunity are not explored in these estimates but are worth considering in future studies when more data become available to differentiate risks at the individual level.

To further understand transmission dynamics and the burden of illness to specific animals, case–control studies for key pathogens to identify specific higher risk animals/settings and risk behaviors facilitating transmission would be beneficial. Additionally, assessment of exposure frequency among Canadians, studies to collect data on pathogen prevalence, concentration and subtyping in relevant animal populations and mechanisms for pathogen reduction for some pets (e.g. reptiles) would further support the understanding of the role animal contact plays in enteric illness transmission and burden.

The burden of enteric infection associated with animal contact is considerable and emphasizes the need for prevention activities. Enhanced awareness and education for the public, farm/occupational workers and pet owners about the potential risk of illness associated with animal contact is key to preventing animal contact-associated illnesses. This includes highlighting the importance of preventative behaviors through consistent messaging at veterinarian offices, pet stores, petting zoos and other venues and reinforcing their role in preventing illness (e.g. recommendations identified in Reducing the Risk of Pet-Associated Zoonotic Infections [3]) and broader communication through websites promoting safe pet ownership, such as the Worms and Germs Blog (http://www.wormsandgermsblog.com) [85] and Healthy Animals, Healthy People [86]. Public health guidance (e.g. petting zoo infection prevention guidelines, recommendations for high-risk populations) and implementation of public health interventions (e.g. enhanced awareness, hand-washing stations) will also contribute to reducing the burden of enteric illness associated with animals. The evaluation of best practices and identification of the most effective prevention activities to reduce disease are required [3].
